# Reduction in the Choking Phenomenon in Elite Diving Athletes Through Changes in Gut Microbiota Induced by Yogurt Containing *Bifidobacterium animalis* subsp. *lactis* BB-12: A Quasi Experimental Study

**DOI:** 10.3390/microorganisms8040597

**Published:** 2020-04-20

**Authors:** Weizhong Dong, Ying Wang, Shuaixiong Liao, Minghang Lai, Li Peng, Gang Song

**Affiliations:** 1 Research Centre For Exercise Detoxification, College of Physical Education, Southwest University, Chongqing 400715, China; dwz123@aliyun.com (W.D.); wangying112233@aliyun.com (Y.W.); lsx199524@swu.edu.cn (S.L.); laiminghang@aliyun.com (M.L.); plmzs@swu.edu.cn (L.P.); 2 Key Lab of Physical Fitness Evaluation and Motor Function Monitoring, College of Physical Education, Southwest University, Chongqing 400715, China

**Keywords:** diving athletes, gut microbiota, choking phenomenon, Bifidobacteriaceae

## Abstract

Objective: The aims of this study are as follows: (1) to understand the relationship between gut microbiota and the choking phenomenon in diving athletes, and (2) to regulate the gut microbiota in diving athletes by drinking yogurt containing *Bifidobacterium animalis* subsp. *lactis* BB-12 and observe changes in the choking phenomenon in diving athletes. Methods: Experiment 1: A total of 20 diving athletes were tested in low- and high-pressure situations. Gut microbiota (*n* = 18) composition was then determined and differences in the gut microbiota composition among diving athletes who presented choking vs. no choking were identified. Experiment 2: A total of 16 divers who presented choking were divided into a high yogurt group (*n* = 6) and a low yogurt group (*n* = 10) for 15 days. Results: (1) The content of Veillonellaceae in divers who presented choking was significantly higher when compared to divers who did not present choking (*p* < 0.05). Bifidobacteriaceae (*r* = −0.52, *p* < 0.05) and Lactobacillaceae (*r* = −0.66, *p* < 0.05) were negatively correlated with the choking index. (2) During experiment 2, the average daily intake of the high yogurt group was 611.78 ± 94.94 mL and the average daily intake of the low yogurt group was 338 ± 71.45 mL and the abundance of Bifidobacteriaceae was significantly higher in the high yogurt group than in the low yogurt group. After the experiment, the choking index in the high yogurt group became significantly lower than that of the low yogurt group (*z* = −3.26, *p* < 0.001). Conclusion: The intake of yogurt containing *B. animalis* subsp. *lactis* can increase the abundance of Bifidobacteriaceae in elite diving athletes and their performance under high pressure. Hence, gut microbiota may affect the choking phenomenon in elite diving athletes.

## 1. Introduction

The avoidance of an abnormal decline in competitive sports performance has been a key focus for high-level athletes preparing for the Olympic Games and is one of the unsolved problems in the field of international sports psychology. In 1984, the sports psychologist Baumeister used the term “choking” to describe the phenomenon of performance decrements [1]. The choking phenomenon is the manifestation of anxiety as an increase in arousal level under perceived pressure, which leads to a serious decline in skilled technical movements [2,3]. In terms of sports, the choking phenomenon is more likely to occur for sports comprising antagonistic and technical actions. The choking phenomenon is the main factor in sports psychology that affects the performance of these kinds of sports. Chinese athletes were traditionally dominant in diving, which is a technical sport. The anxiety of divers during competition may affect them physically when they are performing an action, affecting the connection with the technical action and, subsequently, affecting their performance in the competition [4,5]. At present, there are few reports on the choking phenomenon in elite diving athletes.

At present, the main methods used to interfere with the choking phenomenon are distraction-based interventions and self-focus-based interventions. Distraction-based interventions generally require the implementation of pre-performance routines. Lautenbach et al. used pre-performance routines as an intervention for tennis players and found that, the choking phenomenon in the tennis players was greatly mitigated after the intervention [6]. In terms of self-focus-based interventions, Mesagno et al. investigated high- and low-pressure shot attempts by left-handed and right-handed athletes and the results showed that left-handed and right-handed athletes felt the same amount of pressure, but left-handed athletes performed better than right-handed athletes because of the functional regulation of cerebral hemispheric activity [7]. Through continuous experiments, Beckmann et al. confirmed that athletes undergo specific activation of the cerebral hemisphere under different sporting actions, which can alleviate their performance decline after pressure induction [8]. However, studies involving nutritional intervention have rarely been reported.

There are approximately 10 trillion bacteria in the human intestine that can regulate human health [9]. In 2015, Schmidt reported that the regulation mechanism involves a dialog between the gut and brain, namely, the gut–brain axis. Drinking probiotic yogurt has been shown to reduce women’s responses to pictures of actors with frightened or angry facial expressions [10]. The possible reason for this is that the gut microbiota produce neuroactive compounds, including neurotransmitters and metabolites. These play roles in the brain that are associated with anxiety, depression and other emotional disorders [11]. According to the latest research conducted by Cryan, there is a certain mechanism connecting gut microbiota with social behavior controlled by the brain, and the gut microbiota can also be targeted to affect brain health [12].

Probiotic supplementation has been shown to improve physical and mental health. Akkasheh et al. found that patients with major depressive disorder had lower scores according to the Beck Depression Inventory after probiotic supplementation [13]. Messaoudi et al. found that for healthy people, probiotic intake significantly reduced Hospital Anxiety and Depression Scale (HADS) depression scores during follow-up [14].

There is evidence that gut microbiota can regulate excitatory and inhibitory neurotransmitters (i.e., 5-hydroxytryptamine, γ-aminobutyric acid and dopamine) and neurotransmitter-like substances, especially in response to physical and emotional stress [15]. The choking phenomenon involves sports performance decline caused by external pressure. We hypothesized that the choking phenomenon is related to gut microbiota and that the incidence of the choking phenomenon could be reduced by consuming probiotic (*Bifidobacterium animalis* subsp. *lactis* BB-12) yogurt. Based on this, two consecutive experiments were designed. Experiment 1 was designed to explore the correlation between the gut microbiota and the choking phenomenon in diving athletes. Experiment 2 was designed to observe the possible cause–effect relationship between changes in the gut microbiota and the choking phenomenon under the effect of yogurt containing *B. animalis* subsp. *lactis*. This study aimed to provide a theoretical basis for psychology and physiology by showing that regulation of the gut microbiota can improve the competition performance of athletes under pressure. We applied this to improve the competition performance of divers.

## 2. Materials and Methods

### 2.1. Participants

After medical supervision and inspection, all participants were confirmed to have no organic diseases and normal psychological characteristics. During the experiment, the participants did not take antibiotics. All athletes and their guardians were informed of the whole process of the experiment and provided a signed informed consent before participating in the experiment. This study was approved by the ethics committee of the Southwest University Hospital in Chongqing, China and recorded in the Chinese clinical trial center with the code ChiCTR1900024119.

Experiment 1: 20 divers were recruited for this study. The average age of these athletes was 9.1 ± 1.2 years old, and the average number of training years was 3.75 ± 0.97. All participants ate and lived together. The exclusion criteria were as follows: (1) recent use of antibiotics; (2) occurrence of diarrhea and insomnia in the week before the experiment; and (3) habit of taking supplements containing probiotics. During the collection of fecal samples in Experiment 1, two athletes with the choking phenomenon developed diarrhea. Therefore, the fecal samples of 18 athletes were collected.

Experiment 2: After the completion of Experiment 1, 16 athletes who demonstrated the choking phenomenon were selected to participate in Experiment 2. The average age of the athletes participating in Experiment 2 was 9.19 ± 1.28 years old. Athletes were divided into the high yogurt group (*n* = 6) and the low yogurt group (*n* = 10). In Experiment 2, the training plans of the athletes were recorded, and the athletes were required to avoid intake of other probiotic-containing foods.

### 2.2. Experimental Design

**Figure 1:**
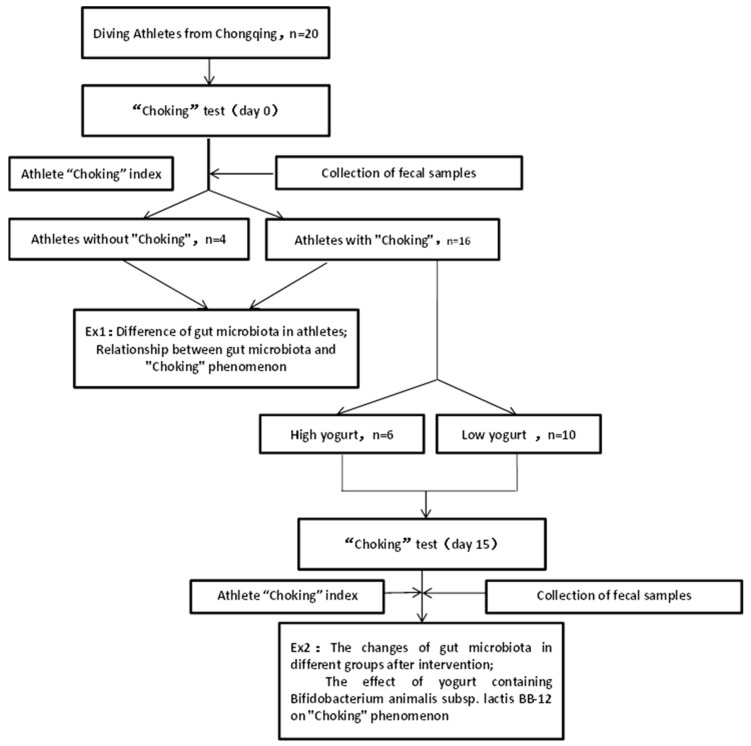
Experimental flowchart.

The whole experiment consisted of two parts. In the first part, we tested the performance of athletes in high- and low-pressure situations as well as characterizing their fecal microbiota contents. In the second part, athletes who demonstrated the choking phenomenon were divided into the high yogurt group and the low yogurt group which determined their yogurt consumption over the next 15 days. Again, we tested the performance of athletes in high- and low-pressure situations as well as characterized their fecal microbiota contents (Figure 1).

### 2.3. Choking Index

Four actions that each athlete must complete in the Youth Diving Championships were selected and used as the basis for evaluating their sports performance. Each athlete was tested twice for each trial. The first test was in the training environment (low-pressure situation), in which only the coaches and athletes were present and there was no audience. The second test was conducted in a man-made competition environment (high-pressure situation). The test environment was a simulated formal competition, and the director of the Chongqing Diving Management Center supervised the competition on the spot. Three referees above the national level were responsible for providing the scores; we also used the parents of these athletes as spectators and broadcast the scores of the athletes on the spot. The scores of these athletes were calculated by the referees (the full score was 10 points, and the average score of the three referees was the final score). Previously, the distance from holes was used as an indicator to measure the phenomenon of choking in golfers [16], and the decline in performance was chosen as an indicator in basketball [17]. Therefore, in the present study, the difference between the high-pressure situation performance score and the low-pressure situation performance score was used as an indicator of the choking phenomenon (choking index). If the choking index score was positive or equal to 0, this was considered to represent no choking phenomenon, while if the difference was negative, this was considered to represent the occurrence of the choking phenomenon. Furthermore, a larger negative value indicated a more obvious choking phenomenon [18].

### 2.4. Yogurt

The yogurt used in the study (Mengniu Dairy (Group) Co., Ltd., Hong Kong, China) is rich in *Bifidobacterium animalis* subsp. *lactis* BB-12 (1 × 10^9^ cfu/100 g), *Lactobacillus bulgaricus* (1 × 10^8^ cfu/100 g), *Streptococcus thermophilus* (1 × 10^8^ cfu/100 g), according to Chinese National Food and Drug Administration domestic health food approval certificate (NO:2015B0306).

The yogurt we bought came in containers of 250 mL, and the yogurt left after drinking was measured. The athletes’ milk boxes were numbered by the staff, and the amount of remaining yogurt in the milk boxes was measured. We used a cream sampler (Beijing Golden Speed Instrument) to take out the remaining yogurt in the bottle and squeezed it into a 25 mL graduated cylinder for measurement. The athlete’s yogurt intake was 250 mL minus this measured value for the remainder. Every day, a researcher was responsible for measuring the daily yogurt intake of athletes.

### 2.5. Gut Microbiota

Sample collection and DNA extraction: Fecal samples were collected at the Affiliated Hospital of Southwest University and frozen at −80 °C within 3 h after sampling. DNA extraction was performed using a QIAamp Fast DNA Stool Mini Kit (Qiagen, California, USA). The concentration of bacterial DNA was measured using a NanoDrop 2000 (Thermo Scientific, Waltham, MA, USA).

High-Throughput Sequencing: The bacterial communities in the fecal samples were investigated by Illumina MiSeq high-throughput sequencing. The V3 and V4 regions of the 16S rDNA gene were selected for PCR. The primers were barcoded as 338F (5′- ACTCCTACGGGAGGCAGCAG-3′) and 806R (5′-GGACTACHVGGGTWTCTAAT-3′), where the barcode was an eight-base sequence that was unique to each sample. The 20 μL PCR reaction mixture was composed of 4 μL of 5× FastPfu buffer, 2 μL of 2.5 mM dNTPs, 5 μM each of forward and reverse primer, 0.4 μL TransStart Fastpfu DNA Polymerase (TransGen Biotech, Beijing, China) and 10 ng DNA template. The following cycling parameters were conducted: maintain at 95 °C for 2 min, 27 cycles (95 °C for 30 s, 55 °C for 30 s and 72 °C for 30 s), and a final extension at 72 °C for 5 min. Triplicate reaction mixtures were pooled for each sample, purified using an AxyPrep DNA gel extraction kit (Axygen, Union City, CA, United States), and quantified using a QuantiFluor-ST Fluorescence quantitative system (Promega, Madison, WI, United States). Amplicons from different samples were sent out for sequencing on an Illumina MiSeq platform at Shanghai Majorbio Bio-Pharm Technology Co., Ltd. (Shanghai, China). The relevant data from the gut microbiota were uploaded to the National Omics Data Encyclopedia (NODE; https://www.biosino.org/node/, Sample ID: OES025599).

Processing of Sequencing Data: Raw fastq files were demultiplexed and quality-filtered using QIIME (version 1.9.1) with the three criteria mentioned in [19]. Operational taxonomic units (OTUs) were clustered with a 97% similarity cutoff using UPARSE (version 7.12), and chimeric sequences were identified and removed using UCHIME. The taxonomy of each 16S rRNA gene sequence was analyzed with the RDP Classifier against the Silva (SSU128) 16S rRNA database by using a confidence threshold of 70%.

### 2.6. Statistical Analysis

A one-sample Kolmogorov–Smirnov test was used to test the normality of the data. The experimental data did not conform to a normal distribution, so a nonparametric test was chosen. The differences in the choking index were analyzed using the Mann–Whitney U-test and the Wilcoxon rank sum test. Spearman’s rank correlation analysis was used to analyze the top 30 families in the gut microbiota and the choking index. Community estimators were calculated and analyzed using Mothur version v.1.30.1 (α-diversity estimators). The Wilcoxon rank sum test was used to measure gut microbiota differences between groups. Benjamini and Hochberg’s false discovery rate (FDR) was used to adjust the results, and significant associations were considered below an FDR threshold of 0.05 (CI bootstrap 0.95). Statistical analysis was carried out using GraphPad Prism V8.3.0 (La Jolla, California, USA) R statistical package (V.3.6.3).

## 3. Results

### 3.1. Experiment 1: The Relationship between Gut Microbiota and the Choking Phenomenon

#### 3.1.1. The Choking Index for Diving Athletes

**Figure 2:**
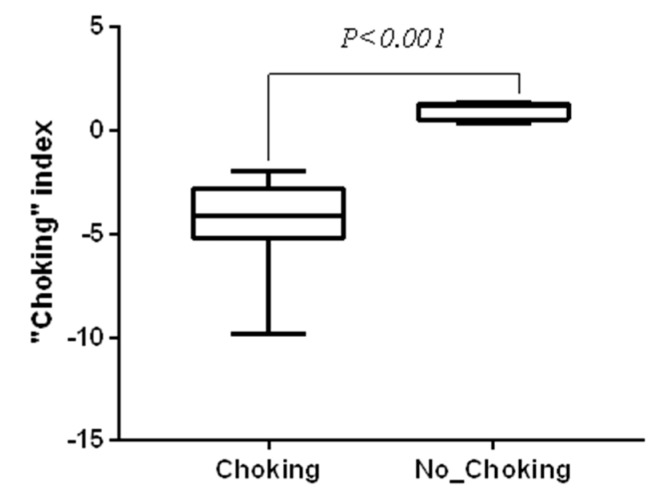
The choking index for diving athletes. Choking index = high-pressure situation performance score−low-pressure situation performance score.

In the present study, four athletes did not present the choking phenomenon (choking index: 1.00 ± 0.45), while 16 athletes did present the choking phenomenon (choking index: −4.76 ± 2.54). The choking index of athletes is shown in Figure 2. There was a significant difference in the choking index between these two groups of athletes (*z* = −3.03, *p* < 0.001).

#### 3.1.2. Differences in Gut Microbiota

**Figure 3:**
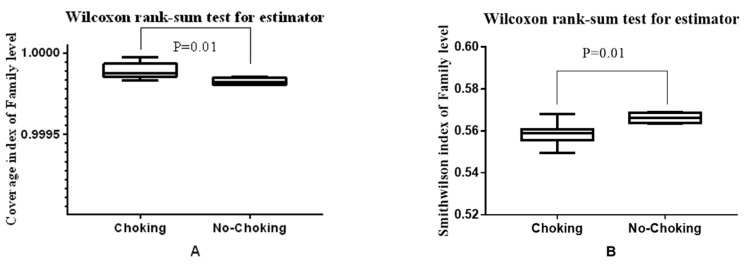
The difference in the α diversity index of athletes with different sports performances is shown. (**A**) is the coverage index, which represents the coverage of the community. (**B**) is the Smith–Wilson index, which represent the evenness of the community.

The results of gut microbiota α diversity index of the athletes with and without choking phenomenon are shown in Figure 3. The results revealed that there were significant differences in the Smith–Wilson index (community evenness, *p* = 0.01) and coverage index (*p* = 0.01) between athletes with and without the choking phenomenon.

**Figure 4:**
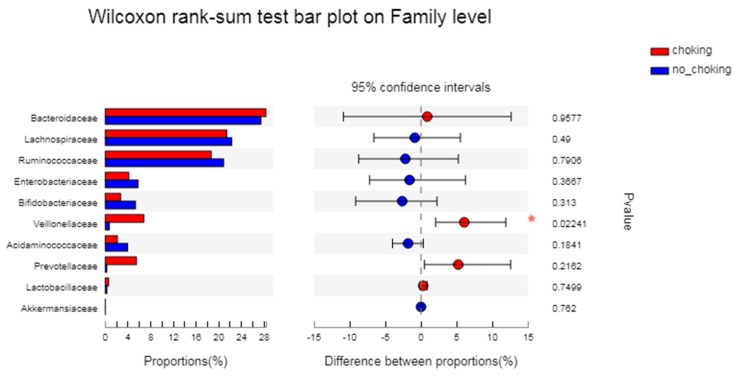
The difference between groups of gut microbiota in athletes with and without the choking phenomenon. The vertical axis (left) shows the name of the bacteria at the family level. Each column corresponding to a family represents the average relative abundance of the family in each sample group. Red represents athletes with the choking phenomenon, while blue represents athletes without the choking phenomenon. The left horizontal axis indicates the proportion of each type of bacteria present. The right horizontal axis indicates the differences between different groups. The middle area shows the differences in family abundance percentage between the two groups within the set confidence interval. The colors of the dots represent the groups with a larger family abundance, and the type I interval of the dot represents the upper and lower limit values of the difference. * *p* < 0.05.

Figure 4 shows that the abundance of Veillonellaceae in athletes with the choking phenomenon was significantly higher than that in athletes without the choking phenomenon (*p* = 0.022).

#### 3.1.3. Correlation Analysis between the Gut Microbiota and Choking Index

Table 1 shows that there is a significant negative correlation between the abundances of Bifidobacteriaceae (r = −0.52, *p* < 0.05) and Lactobacillaceae (r = −0.66, *p* < 0.05) and the choking index. On the other hand, there was a significant positive correlation between Prevotellaceae and the choking index (r = 0.67, *p* < 0.01).

### 3.2. Experiment 2: Yogurt affects the gut microbiota and reduces the choking phenomenon in athletes

#### 3.2.1. Yogurt Intake

**Figure 5:**
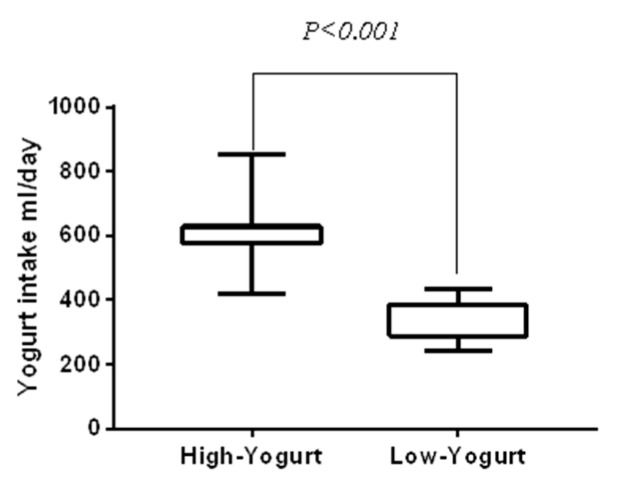
Yogurt intake.

The yogurt intake of athletes during the intervention period is shown in Figure 5. During the 15-day experimental period, the average intake of yogurt was 611.78 ± 94.94 mL per person per day in the high yogurt group, and 338 ± 71.45 mL per person per day in the low yogurt group. The comparison of the yogurt intake between the high yogurt group and low yogurt group revealed that yogurt intake in the high yogurt group was significantly higher than that in the low yogurt group (*z* = −5.827, *p* < 0.001).

#### 3.2.2. The Differences in Gut Microbiota between the High Yogurt Group and Low Yogurt Group

**Figure 6:**
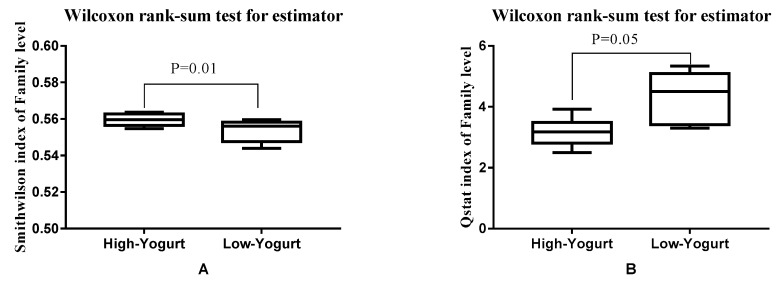
The gut microbiota α diversity index values of athletes in the different groups is shown. (**A**) is the Smith–Wilson index of the two groups of athletes, which reflects the community evenness, while (**B**) presents the qstat index, which reflects the community diversity.

The results of gut microbiota α diversity index of the athletes with different yogurt intake are shown in Figure 6. The results revealed a significant difference between the qstat (community diversity) index (*p* = 0.05) and Smith–Wilson (community evenness) index (*p* = 0.01) values for the gut microbiota in the two groups of athletes in Experiment 2.

**Figure 7:**
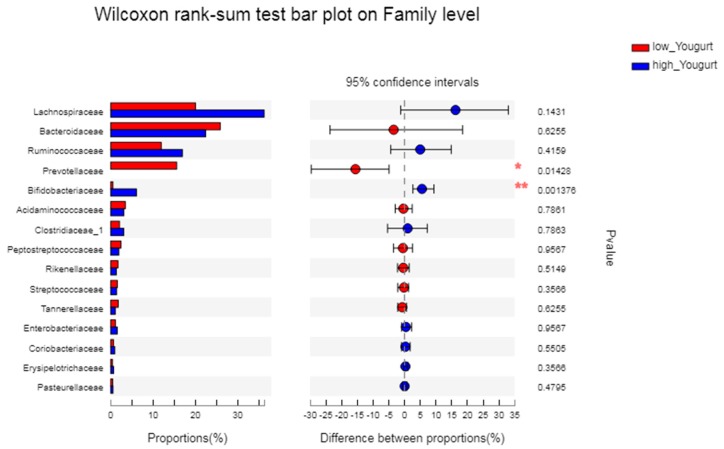
The differences between the groups of gut microbiota are shown. The vertical axis (left) shows the name of the bacteria at the family level. Each column that corresponds to a family represents the average relative abundance of each family in each sample group, and different colors represent the different groups. The middle area represents the difference in abundance percentage between the two groups within the set confidence interval. The colors of the dots represent the groups in which family abundance occupies a large proportion. The I interval on the dot represents the upper and lower limits of the difference. * *p* < 0.05, ** *p* < 0.01.

After comparing the family differences among the different groups of athletes after intervention (Figure 7), it was revealed that the content of Prevotellaceae in the low yogurt group was significantly higher than that in the high yogurt group (*p* = 0.14) and that the content of Bifidobacteriaceae in the high yogurt group was significantly higher than that in the low yogurt group (*p* = 0.001).

#### 3.2.3. The Choking Index of Athletes

**Figure 8:**
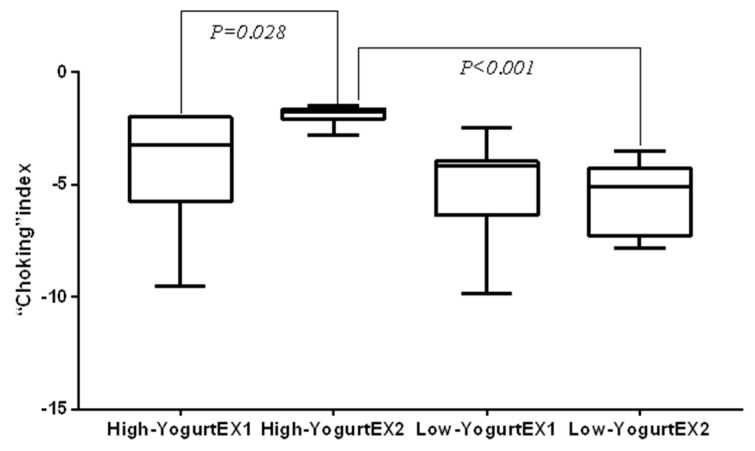
The difference in choking index values between the high and low yogurt groups before and after the test. High-YogurtEX1 and High-YogurtEX2 were assessed with the Wilcoxon signed rank test, while High-YogurtEX2 and Low-YogurtEX2 were assessed with the Mann–Whitney U-test.

Figure 8 shows the difference of choking index between high and low yoghurt groups before and after the test. The Mann–Whitney U-test was performed to assess the choking index in the high and low yogurt groups in Experiment 2. The results revealed that the choking index values in the high yogurt group were significantly lower than those in the low yogurt group (*z* = −3.26, *p* < 0.001). The Wilcoxon rank sum test was used to analyze the choking index values in the high yogurt group in Experiments 1 and 2. The results revealed a significant difference in choking index values between the two groups (*z* = 2.20, *p* = 0.028).

## 4. Discussion

### 4.1. Gut Microbiota and the Choking Phenomenon

Previous studies have focused on the psychological exploration of the formation mechanism of the choking phenomenon. Gao measured the choking index of trampoline athletes and found that 12 of 20 athletes presented choking [18]. The present study found that 16 of the 20 diving athletes presented the choking phenomenon, which was higher than the percentage observed in Gao’s study. The pressure of diving may be greater than that of trampolining, and the choking phenomenon is more likely to occur when under pressure [20]. This shows that there may be differences between sports events in terms of the choking phenomenon.

Studies have shown that there are differences in the α diversity of the gut microbiota between athletes and healthy people, and between exercising people and sedentary people. Research has shown that the diversity of the gut microbiota in male elite professional rugby players is significantly higher than that in healthy males [9,21]. In general college students, a study conducted by Jiang revealed a significant difference in the diversity of Veillonellaceae in the gut microbiota between the less active and more active group [9]. The presence of specific bacteria in the gut can improve sport performance. In July 2019, Scheiman first confirmed that the abundance of *Veillonella* in top marathon runners was significantly higher than that in ordinary people. *Veillonella* obtained from marathon runners were transplanted into mice through fecal microbiota transplantation. It was found that the exercise performance of mice who received the bacterial transplant was better than that of normal mice [22]. The present research revealed significant differences in gut microbiota community evenness and coverage between divers with the choking phenomenon and divers without the choking phenomenon. Further analysis showed a difference in the abundance of Veillonellaceae in athletes with different levels of sports performance, and the abundance of Veillonellaceae in athletes displaying the choking phenomenon was significantly higher than that of divers without the choking phenomenon. Veillonellaceae species help to decompose lactic acid and improve sports performance. In diving, the time from taking off to entering the water is very short, and the main energy supplemental mode uses phosphoric acid as an energy source. Further studies are needed to determine how Veillonellaceae affects performance.

The relationship between the choking phenomenon and gut microbiota has not yet been reported. The correlation analysis revealed that Bifidobacteriaceae and Lactobacillaceae were negatively correlated with the choking phenomenon while Prevotellaceae was positively correlated. This indicates that the choking phenomenon may be improved by probiotics.

### 4.2. Yogurt Containing B. animalis subsp. lactis Reduces the Occurrence of the Choking Phenomenon through Effects on the Gut Microbiota

Intake of yogurt may induce changes in the gut microbiota. The abundance of Bifidobacteria in people who have consumed yogurt for a long time is significantly higher than in people who do not consume yogurt [23]. The abundance of Bifidobacteria in the intestines of healthy adults was shown to increase after drinking yogurt for 30 days [24]. The abundance of Bifidobacteria in individuals significantly increased after 10 days of yogurt intake [25]. In this study, the abundance of Bifidobacteriaceae in the high yogurt group was significantly higher than that in the low yogurt group after 15 days. This shows that yogurt containing *B. animalis* subsp. *lactis* may cause an increase in the abundance of Bifidobacteriaceae in the gut microbiota of elite diving athletes.

Probiotic intake may improve athlete performance. Four weeks of probiotic supplementation reduced gastrointestinal (GI) symptoms during a marathon race and helped athletes to maintain speed during the race [26]. A 12-week experiment revealed that a combination of cycle training and the administration of multiple Bifidobacteria probiotics significantly improved muscle performance [27]. Ralf found that supplementation with probiotics such as Bifidobacteria reduced the concentration of interleukin-6 (IL-6) in the blood after exercise, avoiding a decline in exercise ability and maintaining muscle tone [28]. Furthermore, the supplementation of probiotics such as Bifidobacterium can reduce the plasma endotoxin level in triathlon athletes after competition [29]. Three weeks of probiotic supplementation (*Streptococcus thermophilus* FP4 and *Bifidobacterium breve* BR03) likely enhanced the average isometric peak torque production at 24–72 h into the recovery period following exercise, and selected beneficial bacteria could positively affect athletes undergoing periods of intense training and may assist in performance recovery [28,30].

In this study, yogurt with *B. animalis* subsp. *lactis* induced an increase in the abundance of Bifidobacteriaceae in the bodies of elite diving athletes and decreased the choking index. These changes may be attributed to an increase in the abundance of Bifidobacteriaceae. Bifidobacteria have been shown to reduce stress levels in animal experiments [31], and stress levels can affect the sports performance of athletes under stress [32]. Therefore, one possible explanation is that when athletes drink yogurt that contains probiotics, the abundance of Bifidobacteriaceae increases and athletes feel less pressure. The decrease in pressure enables these athletes to focus on the performance of technical movements, thus improving their performance in a high-pressure situation.

The gut microbiota can directly synthesize more than 90% of 5-hydroxytryptamine (5-HT) and dopamine (DA) in the body [33]. Furthermore, the gut microbiota can regulate excitatory and inhibitory neurotransmitters as well as neurotransmitter-like substances such as 5-HT and DA in response to physical and emotional stress [34]. During tense competition, athletes experience physical and emotional stress. Stress can also affect the microbial composition of the gut by leading to the release of stress hormones or sympathetic neurotransmitters that affect the gut physiology and change the composition of the microbial community [15]. In this study, we made the athletes feel more pressure using a man-made competition situation, and this affected their performance. We found that although the athletes in the high yogurt group still displayed the choking phenomenon, the choking index values changed significantly, and performance under pressure was significantly better than that in the low yogurt group. The results of this study support the hypothesis that yogurt that contains *B. animalis* subsp. *lactis* may improve athlete performance under stress. In addition, it provides some practical experience for ways in which athletes’ gut microbiota and sports performance could be improved through nutrition intervention in the future. However, considering that the relationship between gut microbiota and sports performance is very complex, it cannot be said that the change in gut microbiota is the reason for the change in athlete performance. More research is needed in the future to explore the relationship between gut microbiota and sport performance.

### 4.3. Research Limitations

Due to limitations regarding diver management and sports training, the experimental design could not be randomized.

## 5. Conclusions

In the present study, two consecutive experiments were conducted to explore the effect of the gut microbiota on the occurrence of the choking phenomenon in athletes in high-pressure situations. The results revealed a significant negative correlation between the presence of Bifidobacteriaceae and the occurrence of the choking phenomenon in diving athletes. By drinking yogurt containing *B. animalis* subsp. *lactis*, the content of Bifidobacteriaceae in the gut increased, and the occurrence of the choking phenomenon in athletes was reduced. Probiotic (*B. animalis* subsp. *lactis*) intake may lead to changes in the intestinal health of athletes, improving their perception of stress. When perceived pressure decreased, athletic performance improved. However, more studies are needed to determine the relationship between gut microbiota and the choking phenomenon.

## Figures and Tables

**Table 1:** The correlation between gut microbiota and choking.

	r (Bifidobacteriaceae)	r (Lactobacillaceae)	r (Prevotellaceae)
Choking	−0.52 *	−0.66 **	0.67 **

* *p* < 0.05, ** *p* < 0.01.
